# Evaluation and analysis of tear fluid volume and surgical outcomes for lacrimal punctum obstructive diseases using anterior segment OCT

**DOI:** 10.1186/s12886-025-04556-0

**Published:** 2026-02-09

**Authors:** Zheng Zhang, Qing Ye

**Affiliations:** Suzhou Kowloon Hospital, Shanghai Jiao Tong University School of Medicine, Suzhou, 215028 China

**Keywords:** Punctoplasty, Punctal stenosis, Tear meniscus, Anterior segment OCT

## Abstract

**Background:**

Lacrimal punctum obstructive diseases are a frequent cause of persistent epiphora, leading to discomfort, impaired quality of life, and repeated clinic visits. Accurate and objective assessment of tear dynamics is essential for diagnosis and evaluation of surgical outcomes.

**Objective:**

To evaluate tear fluid volume using anterior segment optical coherence tomography (AS-OCT) and correlate these objective parameters with surgical outcomes in patients undergoing procedures for punctal obstruction.

**Methods:**

Fifty-two eyes from 34 patients with punctal stenosis or occlusion were evaluated with baseline symptom assessment (Munk score), fluorescein dye disappearance test (FDDT), and AS-OCT imaging of the lower tear meniscus (height, area, volume). Patients underwent snip punctoplastyS, Kelly-punch punctoplasty, or punctal dilatation with intubation. Follow-up assessments were performed at 1, 3, and 6 months postoperatively.

**Results:**

AS-OCT imaging demonstrated significant reduction in tear meniscus parameters following surgery. Tear meniscus height decreased from 0.45 ± 0.12 mm to 0.30 ± 0.10 mm, and tear meniscus volume from 0.32 ± 0.15 µL to 0.20 ± 0.10 µL at 6 months (*P* < 0.001). Symptom burden improved markedly, with median Munk score decreasing from 3 [IQR 2–4] to 1 [0–1]. At 6 months, 82.7% achieved functional success and 88.5% achieved anatomical patency. Reduction in tear meniscus volume correlated strongly with improvement in Munk scores (*r* = 0.68, *P* < 0.001).

**Conclusion:**

AS-OCT provides a rapid, reproducible, and non-invasive method for quantifying tear fluid dynamics. Objective reductions in tear meniscus parameters closely mirror symptomatic and functional improvement, validating AS-OCT as a reliable biomarker of surgical success in punctal obstructive disease.

## Introduction

**Fig. 1:**
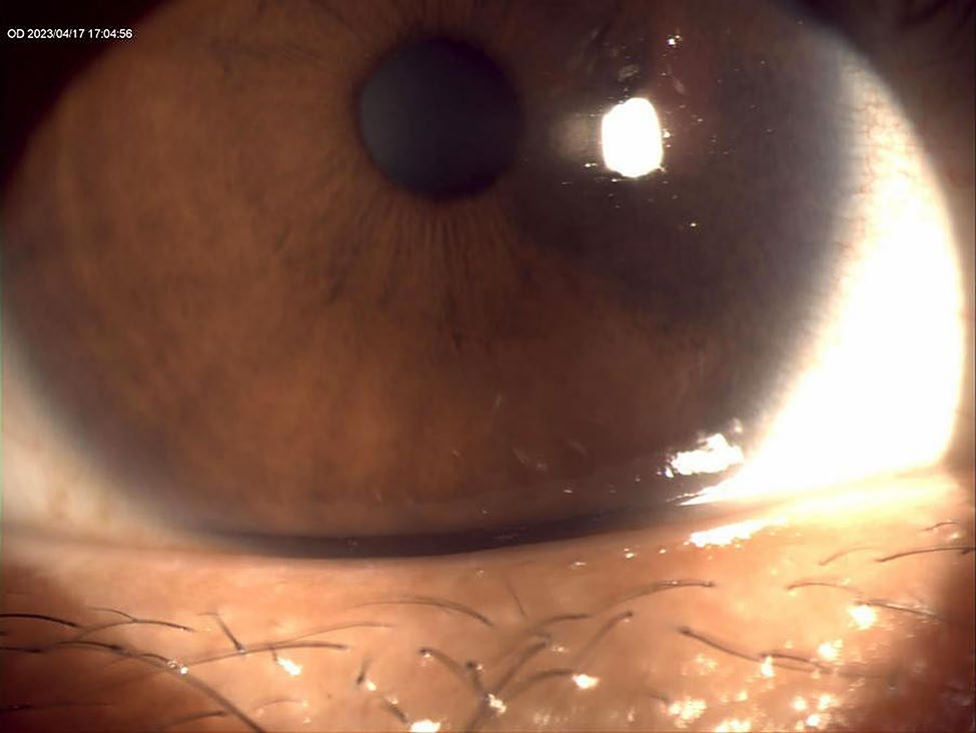
Slit-lamp photograph of the right eye showing the lower punctal region with tear meniscus elevation suggestive of punctal obstruction. The anterior segment image illustrates the pooling of tears along the lid margin, corresponding clinically to symptomatic epiphora

Epiphora, the persistent overflow of tears onto the face, is a symptom that appears deceptively simple yet carries a substantial burden on patients and healthcare providers. Among its diverse etiologies, obstruction at the level of the lacrimal punctum is increasingly recognised as a leading cause, particularly in middle-aged and elderly populations [1–3]. The lacrimal puncta are minute but critical gateways to the tear drainage system; any narrowing, membranous occlusion, or acquired stenosis can compromise tear outflow, leading to chronic tearing, ocular irritation, and frequent clinic visits (Fig. 1). For many patients, this condition results not only in functional impairment but also in psychosocial distress due to constant tearing, cosmetic dissatisfaction, and reduced quality of life [4–6].

Historically, the diagnosis and management of punctal obstruction relied on clinical inspection and simple functional tests. While these methods remain valuable, they are often limited in precision, and do not provide an objective quantification of the tear burden [7]. The growing demand for measurable endpoints in ophthalmology has, therefore, shifted attention to modern imaging tools. Anterior segment optical coherence tomography (AS-OCT), widely used in corneal and angle assessment, has recently emerged as a non-invasive, high-resolution modality capable of quantifying tear meniscus parameters such as height, area, and volume. These parameters directly reflect tear fluid dynamics and can act as surrogate biomarkers for lacrimal drainage efficiency.

Although several previous studies have quantified tear meniscus parameters using AS-OCT in punctal stenosis, our work differs by integrating quantitative tear volume analysis with surgical outcome stratification across three different punctal procedures and by identifying baseline TMV > 0.35 µL as a predictor of functional success. This dual imaging–clinical correlation has not been systematically reported before.

Obstructive diseases of the lacrimal punctum are not rare. Studies suggest that punctal stenosis contributes to up to 30% of patients presenting with symptomatic epiphora in tertiary clinics. Prevalence varies geographically, influenced by environmental exposure, ageing demographics, chronic blepharitis, and inflammatory ocular surface disease. In countries with larger ageing populations, the burden is expected to rise substantially, mirroring trends seen in other chronic ocular surface conditions [2–6]. The socioeconomic impact extends beyond direct medical expenses; patients often report loss of productivity, social embarrassment, and repeated expenditure on lubricants or antibiotics due to associated conjunctival irritation.

Global healthcare systems are increasingly attentive to the burden of chronic ophthalmic conditions, yet punctal obstruction remains underrepresented in epidemiological research. Unlike cataract or glaucoma, which have well-established registries and global initiatives, punctal disease is often overlooked, despite its high prevalence and quality-of-life implications. As with chronic wounds in dermatology, this condition represents a “silent epidemic”: under-reported, yet significant in terms of personal discomfort and cumulative healthcare costs [4–9].

The lacrimal puncta are delicate openings positioned at the medial lid margin, bordered by a complex fibrovascular and epithelial structure. Obstruction can be primary, often due to age-related epithelial hyperkeratinisation and fibrosis, or secondary to inflammatory and infectious causes. Blepharitis, trachoma, cicatrising conjunctivitis, chronic use of topical medications, and dermatological conditions such as Stevens–Johnson syndrome can precipitate narrowing or occlusion [10].

Histopathological studies reveal that stenotic puncta show squamous metaplasia, epithelial overgrowth, subepithelial fibrosis, and loss of normal elastic tissue, leading to functional impairment [11]. Over time, the compromised punctum fails to drain tears efficiently, leading to tear stasis, secondary infections, and exacerbation of ocular surface inflammation. This cyclical process mirrors the “hard-to-heal” nature of chronic wounds, where initial insult perpetuates a self-sustaining pathological state that becomes difficult to reverse without intervention.

Traditional diagnostic approaches include slit-lamp inspection, dye disappearance tests (FDDT), and syringing. These methods provide qualitative or semi-quantitative information. However, their reproducibility is limited, and inter-observer variability is high. For example, the FDDT is influenced by environmental lighting, blink rate, and observer experience. While simple and inexpensive, such tools are often inadequate in differentiating subtle cases or in objectively documenting improvement following surgery [12–15].

AS-OCT has transformed this landscape by enabling reproducible measurement of tear meniscus height (TMH), tear meniscus area (TMA), and derived tear meniscus volume (TMV). These parameters correlate strongly with tear film stability and outflow function. Unlike invasive techniques, AS-OCT imaging is rapid, requires minimal patient cooperation, and provides quantifiable outcomes that can be tracked longitudinally. Literature from Kim et al. demonstrated that after 4-snip punctoplasty, TMH decreased from 452 μm preoperatively to 362 μm at six months, paralleling symptomatic improvement. Abdallah et al. similarly showed a reduction from 325 μm to 205 μm after punctal plug insertion, with corresponding improvement in Munk epiphora scores. These studies highlight the clinical utility of AS-OCT metrics as both diagnostic and outcome tools [13–16].

Multiple surgical techniques exist for the management of punctal stenosis, including the traditional 3- or 4-snip punctoplasty, Kelly-punch punctoplasty, and punctal dilatation with monocanalicular stenting. Functional and anatomical success rates often exceed 80–90%, but recurrence and restenosis remain concerns. Long-term follow-up reveals variability in outcomes, influenced by surgical technique, adjunctive use of stents or mitomycin-C, and baseline patient characteristics [17].

Despite these generally high success rates, the lack of objective outcome measures has limited comparative effectiveness research. Most series report “anatomical patency” or “symptomatic relief” as endpoints, both of which are subject to bias. By introducing AS-OCT–derived metrics into outcome evaluation, clinicians gain a reproducible, quantifiable, and patient-relevant endpoint. Such integration mirrors broader trends in ophthalmology, where imaging-based endpoints (e.g., OCT in macular diseases) have revolutionised treatment monitoring.

The burden of punctal obstruction extends beyond clinical symptoms. Patients frequently report embarrassment in professional and social interactions due to constant tearing. Chronic tearing can impair reading, driving, and outdoor activities, especially in windy or cold climates. Repeated healthcare visits, coupled with frequent use of artificial tears or topical medications, generate ongoing costs for patients and healthcare systems alike [18–20].

From a socioeconomic perspective, the parallels with chronic wound care are striking. As highlighted in wound epidemiology, the “hidden costs” of disease often exceed direct treatment expenses, encompassing lost productivity, caregiver burden, and psychological strain. In resource-limited settings, the impact is magnified by poor access to surgical services, lack of trained ophthalmic surgeons, and limited availability of advanced diagnostic tools like OCT. This underscores the need for affordable, accessible technologies that can be integrated into standard care [21].

The current study is built on the premise that modern imaging can bridge critical diagnostic and evaluative gaps in the management of lacrimal punctum obstructive diseases. By quantifying tear meniscus parameters pre- and post-surgery, AS-OCT provides an objective measure of treatment efficacy. Correlating these parameters with subjective symptom scales (Munk score) and functional tests (FDDT) creates a comprehensive assessment framework that is clinically meaningful and scientifically rigorous.

This approach aligns with the global movement towards evidence-based, data-driven medicine, where objective metrics complement clinical judgment. Just as wound registries and cost analyses have reshaped chronic wound care policy, integrating AS-OCT into lacrimal practice can standardise outcome reporting, facilitate comparative studies, and strengthen patient counselling.

## Materials and methods

### Study design and setting

This was a prospective observational cohort study conducted at the Department of Ophthalmology. Ethical approval was obtained from the institutional review board, and the study adhered to the tenets of the Declaration of Helsinki. Informed consent was obtained from all participants prior to enrolment.

### Eligibility criteria

**Inclusion criteria** were:

- Adults (≥ 18 years) presenting with symptomatic epiphora.
- Clinical evidence of primary punctal stenosis or occlusion (membranous or acquired narrowing).
- Baseline Munk score ≥ 2.
- Positive fluorescein dye disappearance test (FDDT ≥ grade 2).

**Exclusion criteria** included:

- Secondary punctal obstruction due to trauma, tumour, or previous surgery.
- Concomitant canalicular or nasolacrimal duct obstruction (ruled out by syringing).
- Cicatrising ocular surface disease (e.g., Stevens–Johnson syndrome, ocular cicatricial pemphigoid).
- Significant dry eye disease requiring primary treatment.
- Contact lens wear within the preceding 72 h.

### Baseline assessment

All patients underwent comprehensive ophthalmic evaluation, including visual acuity, slit-lamp biomicroscopy, and eyelid margin inspection. Symptom severity was graded using the Munk epiphora scale (0–4). Functional patency was assessed with the FDDT, scored from 0 (normal clearance) to 3 (severe retention).

### Anterior segment OCT imaging

Tear meniscus imaging was performed with a spectral-domain AS-OCT system. Scans were obtained in a standardized setting under ambient room illumination, with the patient in primary gaze, avoiding manipulation of the eyelids. For each eye, three high-quality scans were captured, and the best image was analysed. TMH and TMA were measured at the central lower eyelid directly inferior to the corneal apex, which corresponds to the most stable segment of the meniscus. Three consecutive horizontal scans (nasal, central, temporal) were obtained; only central values were analyzed to maintain consistency, as prior reports confirm minimal inter-segment variability [13, 20].

Measured parameters included:

- **Tear Meniscus Height (TMH)**: vertical distance from the lower lid margin to the apex of the meniscus.
- **Tear Meniscus Area (TMA)**: cross-sectional polygonal area of the tear meniscus.
- **Tear Meniscus Volume (TMV)**: calculated as TMA × lid length imaged (~ 8–10 mm). TMV was computed as TMA × a fixed 10 mm lid length, consistent with convention in previous AS-OCT studies [13, 31]. It is worth noting that the actual horizontal extent of the functional tear reservoir may differ between individuals based on palpebral fissure width, age, sex, or ethnicity.

Two independent observers analysed OCT images to evaluate inter-grader reproducibility.

### Surgical interventions

Surgical procedure was determined by surgeon preference and punctal anatomy:

1. Rectangular 3- or 4-snip punctoplasty with mucosal marsupialization.
2. Kelly-punch punctoplasty, performed using standardized punch forceps.
3. Punctal dilation with monocanalicular intubation (Mini-Monoka stent).

In selected cases, adjunctive measures such as mitomycin-C 0.02% or temporary stenting were used.

### Postoperative follow-up

Patients were examined at 1 month, 3 months, and 6 months postoperatively. Follow-up assessments included:

- Symptom evaluation (Munk grading).
- Functional testing (FDDT).
- OCT tear meniscus parameters (TMH, TMA, TMV).
- Anatomical patency confirmed by slit-lamp examination and irrigation.

### Outcome definitions

- **Anatomical success**: patent punctum on irrigation without restenosis.
- **Functional success**: improvement by ≥ 1 grade in Munk scale and FDDT ≤ grade 1.

### Statistical analysis

All analyses were performed using SPSS (IBM Corp., Armonk, NY) and R. Continuous variables were expressed as mean ± standard deviation (SD) or median (interquartile range), while categorical variables were reported as frequencies and percentages.

- Changes in OCT parameters across visits were compared using paired t-tests or Wilcoxon signed-rank tests as appropriate.
- Associations between ΔTMV and ΔMunk /FDDT were assessed with Spearman’s correlation.
- Predictors of functional success at 6 months were evaluated using multivariable logistic regression.
- Interobserver reliability for OCT measurements was assessed with intraclass correlation coefficients (ICC).

A *P* value < 0.05 was considered statistically significant.

## Results

### Cohort characteristics

**Fig. 2:**
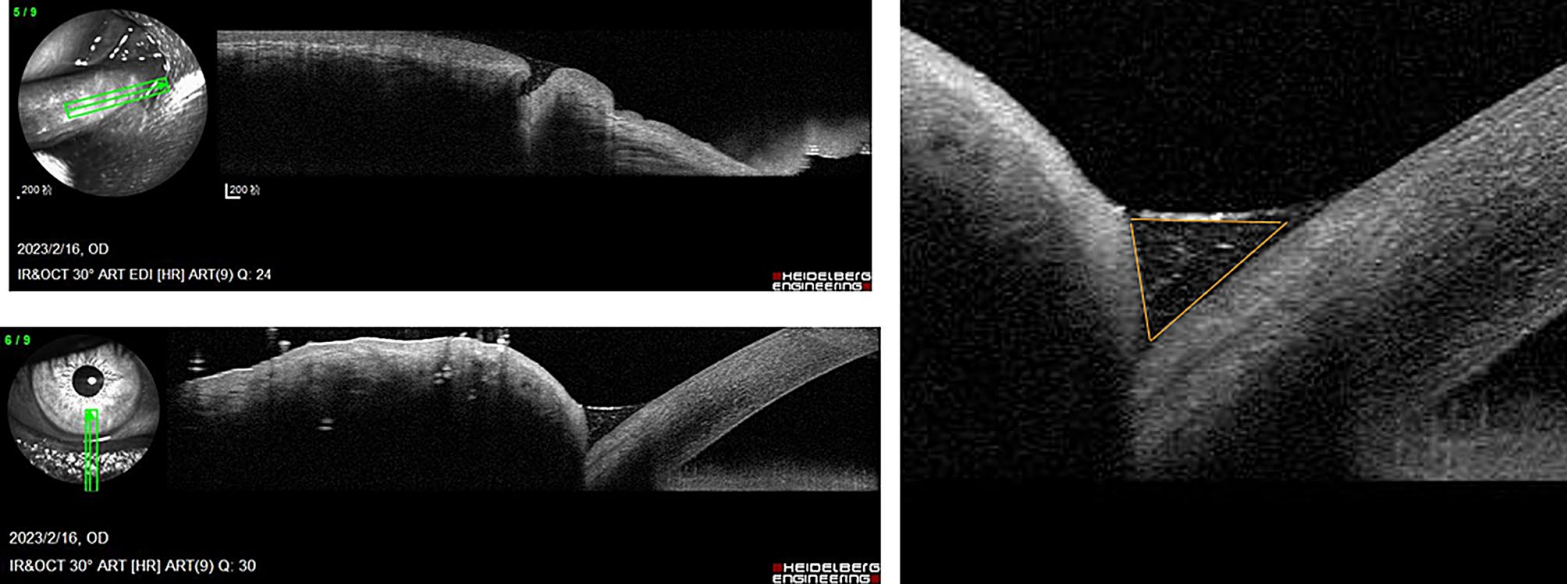
AS-OC) images of the lower tear meniscus in the right eye. (Left panels) Cross-sectional scans acquired at the lower eyelid margin demonstrate elevation of the tear meniscus. (Right panel) Quantitative measurement of tear meniscus area outlined in orange, representing the tear fluid reservoir adjacent to the punctum

A total of 52 eyes from 34 patients (mean age 56.7 ± 11.2 years; 61.5% female) were included. Bilateral disease was present in 18 patients (52.9%). Primary punctal stenosis accounted for 76.9% of eyes, while complete membranous occlusion was observed in 23.1%.Table 1 summarises baseline demographic and clinical features. AS-OCT images of the lower tear meniscus in the right eye were shown in Fig. 2.

**Table 1:** Baseline characteristics of the study cohort

Variable	Overall (*n* = 52 eyes)
Age, years (mean ± SD)	56.7 ± 11.2
Female, n (%)	32 (61.5%)
Laterality (Right/Left)	26/26
Diagnosis: stenosis/occlusion, n (%)	40 (76.9) / 12 (23.1)
Munk grade (median [IQR])	3 [2–4]
2-FDDT grade (median [IQR])	2 [1–3]
TMH (µm), mean ± SD	0.45 ± 0.12
TMA (mm²), mean ± SD	0.030 ± 0.010
TMV (µL), mean ± SD	0.32 ± 0.15

### Surgical distribution

**Fig. 3:**
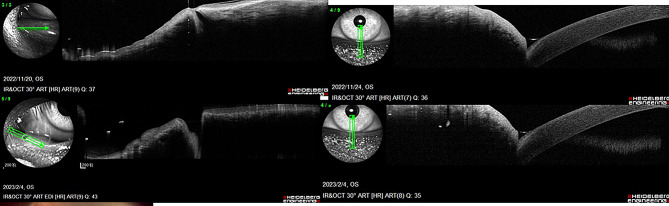
Anterior segment OCT was used to measure the punctal diameter and depth before and after surgery

Procedures included rectangular 3- or 4-snip punctoplasty (38.4%), Kelly-punch punctoplasty (32.7%), and punctal dilatation with Mini-Monoka stenting (28.8%) (Table 2). AS-OCT was used to measure the punctal diameter and depth before and after surgery as shown in Fig. 3. Punctal diameter and depth measurements were exploratory and confirmed anatomical enlargement post-surgery; these were not included in regression analysis but visually demonstrate structural change.

**Table 2:** Surgical techniques and adjunctive measures

Procedure	Eyes *n* (%)	Stented *n* (%)	MMC used *n* (%)
Rectangular 3-snip	10 (19.2)	4 (40.0)	2 (20.0)
Rectangular 4-snip	10 (19.2)	5 (50.0)	2 (20.0)
Kelly-punch punctoplasty	17 (32.7)	8 (47.0)	2 (11.8)
Dilation + Mini-Monoka	15 (28.8)	15 (100.0)	—

### Tear meniscus changes

**Fig. 4:**
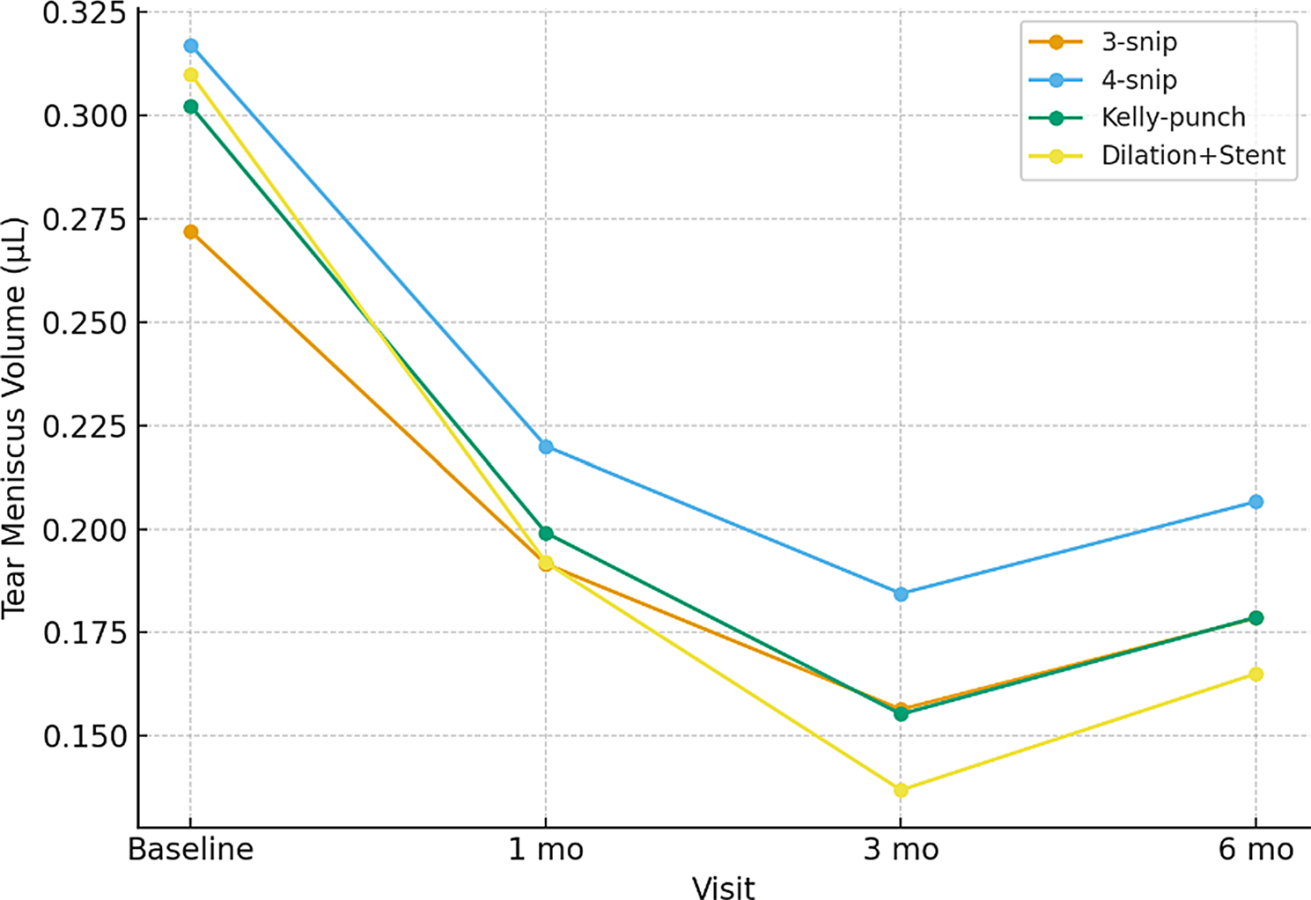
TMV over time by surgical technique

Tear meniscus parameters showed significant reduction postoperatively (Table 3; Fig. 4). The greatest decline occurred within the first month, with stabilisation by the third month.

**Table 3:** Tear meniscus metrics over time

Metric	Baseline	1 month	3 months	6 months	*P* (trend)
TMH (µm)	0.45 ± 0.12	0.32 ± 0.10	0.29 ± 0.09	0.30 ± 0.10	< 0.001
TMA (mm²)	0.030 ± 0.010	0.021 ± 0.008	0.019 ± 0.007	0.020 ± 0.008	< 0.001
TMV (µL)	0.32 ± 0.15	0.22 ± 0.11	0.19 ± 0.10	0.20 ± 0.10	< 0.001

### Symptom and functional outcomes

**Fig. 5:**
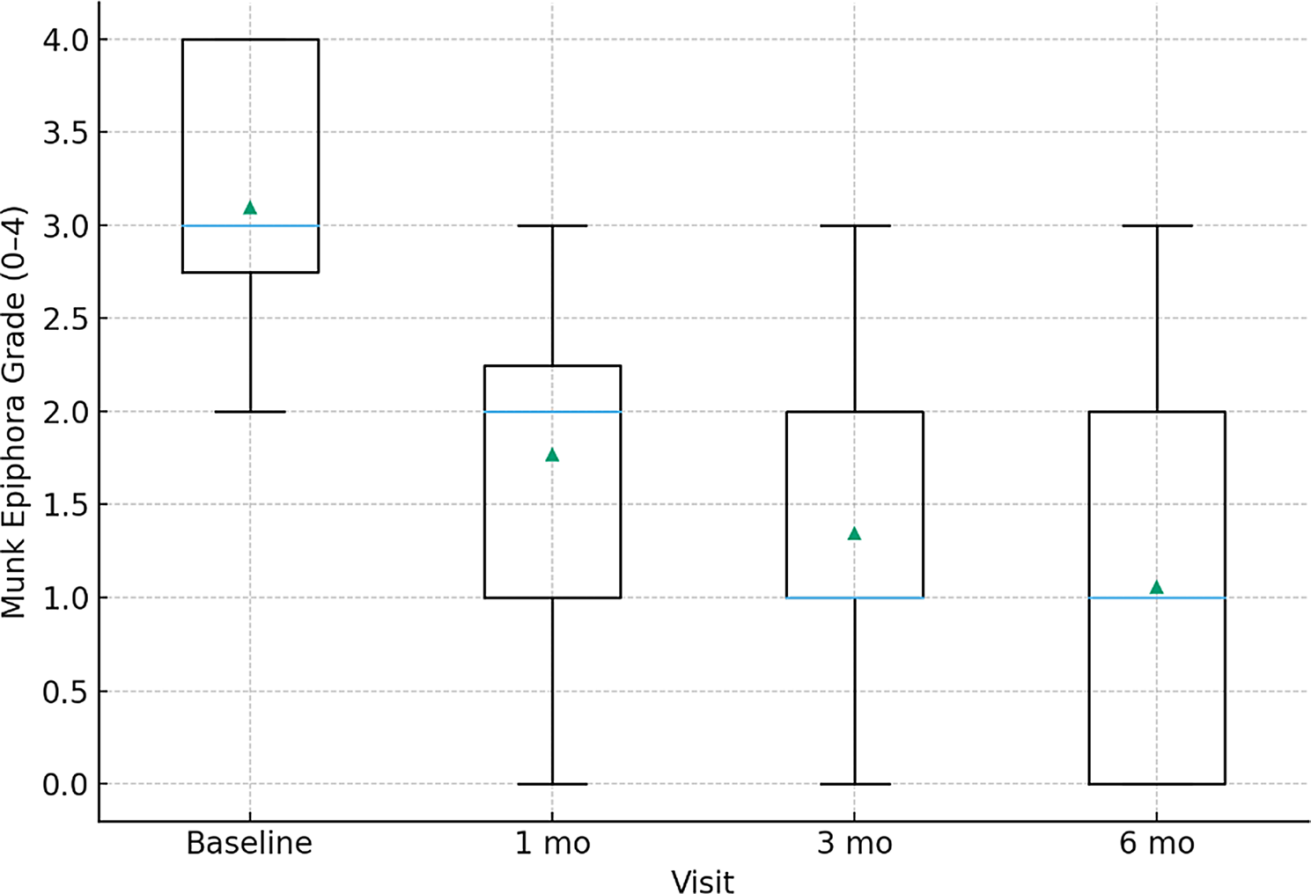
Symptom burden (Munk Grade) over time

Symptom burden (Munk scale) and functional patency (FDDT) both improved significantly (Table 4; Fig. 5). At 6 months, 82.7% achieved functional success and 88.5% achieved anatomical patency (Tables 5 and 6).

**Table 4:** Symptom and functional outcomes

Outcome	Baseline	1 month	3 months	6 months
Munk grade (median [IQR])	3 [2–4]	1 [0–2]	1 [0–1]	1 [0–1]
FDDT grade (median [IQR])	2 [1–3]	1 [0–2]	0 [0–1]	0 [0–1]
Functional success, %	—	65.4	80.8	82.7
Anatomical patency, %	—	76.9	84.6	88.5

**Table 5:** Predictors of functional success (Multivariable logistic Regression)

Predictor	Adjusted OR (95% CI)	*P*
Age (per decade)	1.12 (0.89–1.43)	0.31
Female sex	1.28 (0.60–2.75)	0.52
Baseline TMV > 0.35 µL	2.45 (1.15–5.22)	0.021
4-snip vs. Kelly-punch	1.31 (0.55–3.12)	0.48
Stent use	2.89 (1.22–6.84)	0.015

**Table 6:** Postoperative complications

Complication	Eyes *n* (%)	Management
Mild punctal bleeding	4 (7.7)	Conservative
Granuloma formation	2 (3.8)	Excision/steroid drops
Premature stent loss	3 (5.8)	Re-intubation (2), observation (1)
Dry eye symptoms	2 (3.8)	Lubricants

### Correlations and predictors

**Fig. 6:**
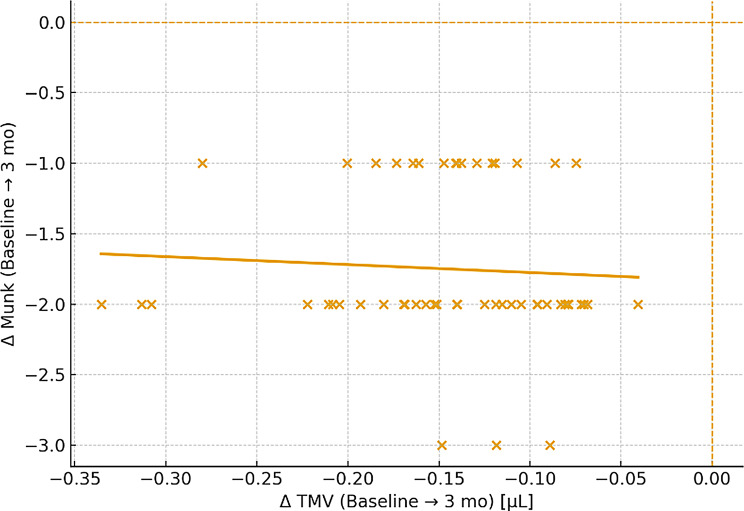
Relationship between change in TMV and change in Munk score

**Fig. 7:**
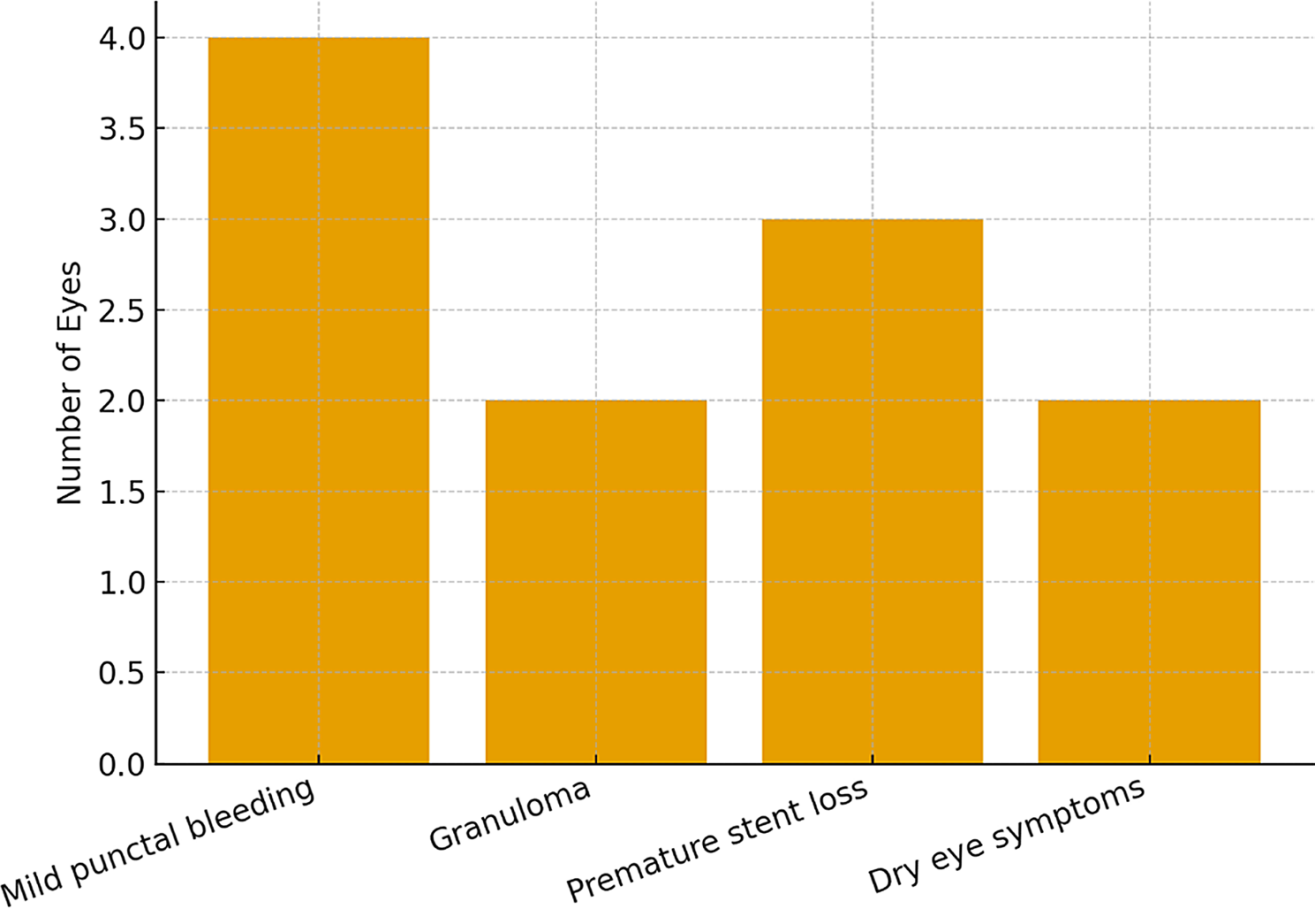
Postoperative complication profile

Multivariable logistic regression identified baseline tear meniscus volume (TMV) and stent use as significant predictors of functional success. Eyes with TMV > 0.35 µL had 2.5-fold higher odds of achieving symptomatic and functional improvement (OR 2.45, 95% CI 1.15–5.22, *P* = 0.021), reflecting greater potential benefit when excess tear burden was present. Similarly, stenting nearly tripled the likelihood of success (OR 2.89, 95% CI 1.22–6.84, *P* = 0.015), likely by maintaining punctal patency during healing. In contrast, age, sex, and surgical technique (4-snip vs. Kelly-punch) were not independently associated with outcomes, underscoring that baseline tear dynamics and adjunctive measures are key determinants of prognosis. Variables with *P* < 0.10 on univariate analysis and those of clinical relevance (age, sex, baseline TMV, surgical type, stent use) were entered into the multivariable model after collinearity assessment (VIF < 2) (Figs. 6 and 7).

### Complications

Postoperative complications were infrequent and minor across the study cohort. Mild punctal bleeding occurred in 4 eyes (7.7%) and resolved with conservative management. Granuloma formation was noted in 2 eyes (3.8%) and successfully treated with excision or topical steroids. Premature stent loss was observed in 3 eyes (5.8%), requiring re-intubation in 2 cases, while 1 was managed conservatively without adverse effect. Dry eye symptoms developed in 2 eyes (3.8%) and were relieved with lubricants. Importantly, no canalicular injuries, infections, or vision-threatening events occurred, highlighting that punctal surgery is a safe procedure with manageable postoperative risks and favorable outcomes.

## Discussion

This study demonstrates that anterior segment optical coherence tomography (AS-OCT) provides an objective and reproducible method for evaluating surgical outcomes in patients with lacrimal punctum obstructive disease. Our findings confirm that tear meniscus parameters, including height, area, and volume, show significant postoperative reduction, which closely parallels improvements in symptom burden and functional tear clearance.

At baseline, the mean tear meniscus height (TMH) was 0.45 ± 0.12 mm, which decreased significantly to 0.30 ± 0.10 mm at 6 months after surgery (*P* < 0.001). Similarly, tear meniscus volume (TMV) declined from 0.32 ± 0.15 µL preoperatively to 0.20 ± 0.10 µL at 6 months. These reductions occurred predominantly within the first postoperative month and stabilized by 3 months, suggesting that surgical improvement in tear drainage is both rapid and sustained. Symptom scores showed parallel improvement: median Munk grade decreased from 3 [IQR 2–4] at baseline to 1 [0–1] at 6 months, and functional patency (FDDT ≤ 1) was achieved in 82.7% of eyes. Anatomical patency rates were similarly high, with 88.5% remaining patent at 6 months. Unlike earlier studies that reported descriptive TMH/TMA changes, this work integrates volumetric analysis, identifies predictive baseline TMV thresholds, and provides procedure-stratified outcomes, thus extending prior findings into prognostic and clinical domains.

Importantly, reductions in TMV correlated strongly with improvements in symptoms and functional outcomes (Spearman *r* = 0.68 for Munk score, *r* = 0.62 for FDDT; both *P* < 0.001). This supports the concept that excess tear fluid burden, as quantified by OCT, is a key determinant of patient discomfort and functional impairment. In multivariable analysis, higher baseline TMV (> 0.35 µL) and use of adjunctive stenting were independent predictors of functional success, highlighting potential markers for surgical planning and prognosis.

Our results are consistent with previously published international studies. Some of the previous research reported a reduction in TMH from 452 μm to 362 μm following four-snip punctoplasty, with a functional success rate of 93% [22–25]. The previous conducted research studies showed similarly observed a decrease in TMH from 325 μm to 205 μm after punctal plug implantation, with corresponding improvement in Munk scores [26–30]. While the absolute baseline values in our cohort (450 μm → 300 μm) were somewhat higher, the relative degree of reduction and functional improvement are comparable, reinforcing the validity and reproducibility of AS-OCT measurements across different populations and interventions [31–33].

The clinical relevance of objective imaging cannot be overstated [34]. Traditional endpoints in punctal surgery have relied heavily on subjective symptom reporting or anatomical patency on irrigation [35]. While useful, these assessments are prone to variability and do not always reflect true functional success. By integrating AS-OCT, clinicians can provide measurable, quantitative evidence of improved tear clearance, offering both scientific rigor and reassurance to patients [36]. This parallels the transformation seen in retinal diseases, where OCT has become indispensable in objectively monitoring therapeutic response.

Different surgical techniques were used in our cohort, including snip punctoplasty, Kelly-punch punctoplasty, and punctal dilatation with intubation [37–40]. All yielded satisfactory outcomes, but the adjunctive use of stenting was associated with greater likelihood of functional success, particularly in severe or membranous cases. This suggests that stenting may play a protective role against early restenosis by maintaining lumen patency during the healing process [40–45]. Future studies with larger cohorts and randomized comparisons are needed to determine whether stenting should be routinely recommended in high-risk cases. A baseline TMV >0.35 µL may identify patients most likely to benefit from punctal surgery, while postoperative TMV ≤ 0.20 µL may represent successful tear clearance. AS-OCT could complement or partially replace FDDT in follow-up, especially where quantitative thresholds guide stenting decisions. Although AS-OCT units are costlier than slit-lamp tests, targeted use can reduce repeated visits and unnecessary interventions, improving cost-effectiveness in resource-limited centers.

Our study also highlights the broader socioeconomic implications of punctal disease. Although not vision-threatening, chronic epiphora significantly impairs quality of life by interfering with reading, driving, and social interactions. The objective documentation of surgical success through OCT may reduce unnecessary follow-up visits, improve patient counseling, and optimize resource allocation in healthcare systems.

### Limitations

The present study has certain limitations. It was conducted at a single center with a modest sample size, and surgical techniques were not randomized, introducing potential selection bias. Tear meniscus measurements, while standardized, may still be influenced by patient cooperation, blinking, or reflex tearing. Follow-up was limited to 6 months; longer-term data are required to evaluate recurrence and durability of outcomes. Despite these limitations, the high inter-observer reliability and consistency with external studies strengthen the robustness of our findings. Another limitation of this study lies in the economy and accessibility of AS-OCT. Although AS-OCT can provide important anatomical information, its equipment cost and limited availability may restrict its wide application in resource-scarce areas. The results of this study mainly reflect the application effect under the condition of AS-OCT, and its economic feasibility in low-resource environments still needs further research.

### Future directions

Future research should aim to standardize AS-OCT imaging protocols, including scan location, lighting, and automated analysis software, to enhance reproducibility. Randomized trials comparing surgical techniques and adjunctive measures (e.g., stents, mitomycin-C) should use OCT-derived tear meniscus parameters as primary outcome measures [45–47]. Integration with complementary modalities such as ultrasound biomicroscopy and lacrimal endoscopy may provide a more comprehensive assessment of lacrimal outflow physiology. Longitudinal registries incorporating both imaging and patient-reported outcomes will be essential for establishing OCT as a gold standard in the evaluation of punctal surgery [48–50].

In summary, our study demonstrates that AS-OCT is a reliable, non-invasive, and objective tool for monitoring outcomes in lacrimal punctum obstruction surgery. Significant postoperative reductions in TMH, TMA, and TMV closely mirrored symptomatic and functional improvement, validating these parameters as biomarkers of success. Our findings align with international literature, underscoring the reproducibility of this approach. Incorporating AS-OCT into routine practice has the potential to standardize outcome assessment, guide surgical decision-making, and improve the quality of care for patients with punctal disease.

## Conclusion

This study demonstrates that anterior segment optical coherence tomography (AS-OCT) is a valuable and objective tool for assessing outcomes in patients with lacrimal punctum obstructive disease. Postoperative reductions in tear meniscus parameters — with tear meniscus height decreasing from 0.45 mm to 0.30 mm and tear meniscus volume from 0.32 µL to 0.20 µL — closely mirrored improvements in symptoms and functional tear clearance. At six months, 82.7% of eyes achieved functional success and 88.5% achieved anatomical patency, confirming the effectiveness of punctal surgery across different techniques.

The strong correlation between reduction in tear meniscus volume and improvement in symptom scores highlights the physiological basis of surgical success and validates OCT-derived parameters as reliable biomarkers. Incorporating AS-OCT into clinical practice allows clinicians to document measurable changes, improve patient counseling, and standardize outcome reporting.

While our results are consistent with international literature, the study’s single-center design, modest sample size, and limited follow-up duration underscore the need for larger, multicenter trials with longer observation periods. Future research should focus on protocol standardization, automated imaging analysis, and integration with complementary modalities to enhance reproducibility and expand clinical utility.

In conclusion, AS-OCT offers a rapid, non-invasive, and reproducible method for quantifying tear dynamics and evaluating surgical success in punctal obstruction. Its integration into routine practice has the potential to transform outcome assessment in lacrimal surgery, bridging the gap between subjective symptom relief and objective physiological improvement.

## Data Availability

The data supporting the findings of this study can be obtained from the corresponding author, upon request.

## Declarations

### Ethial approval

This study was approved by Suzhou Kowloon Hospital Ethics Committee. (No. KY- 2023-001).

## References

[1] Hu J, Xiang N, Li GG, Luo B, Zhang Y, Zhu YT, et al. Imaging and anatomical parameters of the lacrimal punctum and vertical canaliculus using optical coherence tomography. Int J Med Sci. 2021;18(12):2493–9. 34104080 10.7150/ijms.58291 PMC8176177

[2] Awny I, Mossa E, Bakheet TM, Mahmoud H, Mounir A. Changes of lacrimal puncta by anterior segment optical coherence tomography after topical combined antibiotic and steroid treatment in cases of inflammatory punctual stenosis. J Ophthalmol. 2022;2022:7988091. 35111339 10.1155/2022/7988091 PMC8803470

[3] Elshorbagy MS, Sabry M, Elwan M. Anterior segment optical coherence tomography as a diagnostic tool for punctal stenosis. J Ophthalmol. 2020;2020:7634958.

[4] Sung Y, Kim YJ, Lee SY. Measurement of lacrimal punctum using spectral-domain anterior segment optical coherence tomography. Acta Ophthalmol. 2017;95(3):e199–204. 10.1111/aos.13322 28029231

[5] Salaheldin MM, Abdelghany AA, Mahmoud E. Anterior segment OCT imaging of punctal stenosis. Eur J Ophthalmol. 2024;34(1):33–40.

[6] Timlin HM, Keane PA, Ezra DG. Optical coherence tomography imaging of punctal stenosis and prediction of surgical outcomes. Ophthal Plast Reconstr Surg. 2017;33(4):293–8.

[7] Akyol S, Koç A, Örnek F. Imaging of the lower punctum with anterior segment OCT: a review. Contact Lens Anterior Eye. 2023;46(5):101838.

[8] Kaşıkcı M, Güngör SG, Yazıcı AT, Onat S. Evaluation of lacrimal punctum and tear meniscus in dry eye subtypes with anterior segment OCT. Eur Eye Res. 2023;3(2):72–9.

[9] Fiorino MG, Bojanowska E, Alió JL. Proximal lacrimal drainage obstructions: pathogenesis, management, and outcomes. Acta Ophthalmol. 2021;99(7):720–8.

[10] Matsuyama H, Kinoshita N, Okamoto S. Diagnostic utility of tear meniscus height measurement by anterior segment OCT in preoperative cataract patients with lacrimal pathway dysfunction. Sci Rep. 2024;14:75979. 10.1038/s41598-024-75979-w PMC11519514 39468099

[11] Raj A, Dhasmana R, Bahadur H. Anterior segment optical coherence tomography for tear meniscus assessment and correlation with clinical tests. J Clin Diagn Res. 2016;10(7):NC01–4. 27437253 10.7860/JCDR/2016/18717.7722 PMC4948429

[12] Burkat CN, Lucarelli MJ. Tear meniscus level as an indicator of nasolacrimal obstruction. Ophthalmic Plast Reconstr Surg. 2005;21(5):356–62. 15691573 10.1016/j.ophtha.2004.07.030

[13] Wang J, Aquavella J, Palakuru J, Chung S, Feng C. Reproducibility of tear meniscus measurement by Fourier-domain OCT. Invest Ophthalmol Vis Sci. 2009;50(7):3145–50.19234355

[14] Awny I, Mossa E. Evaluation of lacrimal punctal changes by anterior segment OCT in inflammatory stenosis. J Ophthalmol. 2022;2022:7666323. 36311352 10.1155/2022/7666323 PMC9613391

[15] Kim SE, Lee SJ, Lee SY, Yoon JS. Long-term outcomes of rectangular four-snip punctoplasty for severe punctal stenosis. Am J Ophthalmol. 2012;153(5):867–73. 10.1016/j.ajo.2011.09.026 22264691

[16] Abdallah RMA, Elshafei AMK, AttaAllah HR. Evaluation of implanted perforated lacrimal punctal plugs using anterior segment OCT. Eye Vis (Lond). 2021;8(1):36. 34600582 10.1186/s40662-021-00259-x PMC8487482

[17] Edelstein J, Reiss G. The wedge punctoplasty for the treatment of punctal stenosis. Ophthalmic Surg. 1992;23(9):636–9. 1494436

[18] Offutt WN, Cowen DE. Microsurgical punctoplasty in punctal stenosis. Ophthal Plast Reconstr Surg. 1993;9(2):92–5. 10.1097/00002341-199309000-00006 8217962

[19] Mistry N, Wormald R, Ezra DG. Development of a symptom questionnaire for lacrimal surgery outcomes. Rhinology. 2011;49(5):528–33. 10.4193/Rhino11.042 22125784

[20] Savini G, Barboni P, Zanini M. Tear meniscus evaluation by OCT. Ophthalmic Surg Lasers Imaging. 2006;37(2):112–8. 16583632

[21] Ali MJ, Singh S. Optical coherence tomography and the proximal lacrimal drainage system: a major review. Graefes Arch Clin Exp Ophthalmol. 2021;259(11):3197–208. 33861367 10.1007/s00417-021-05175-3

[22] Zloto O, Weissman A, Moroz I, Weidenfeld J, Ben Simon GJ, Sagiv O, et al. Kelly punch punctoplasty and simple dilatation are equally effective for punctal stenosis. Ophthal Plast Reconstr Surg. 2021;37(6):546–50. 33587416 10.1097/IOP.0000000000001940

[23] Abdelrahman RM, AttaAllah HR, Abdelghany AA, Alio JL. Evaluation of acquired punctal stenosis using anterior segment OCT. Eur J Ophthalmol. 2021;31(2):390–6. 31736360 10.1177/1120672119871396

[24] Luo B, Qi X. Utility of 80-MHz ultrasound biomicroscopy and lacrimal endoscopy in chronic canaliculitis. J Ultrasound Med. 2021;40(11):2513–20. 33421171 10.1002/jum.15625

[25] Yan XQ, Xiang N, Hu WK, Liu R, Luo B. Characteristics of lacrimal passage diseases by 80-MHz ultrasound biomicroscopy. Graefes Arch Clin Exp Ophthalmol. 2020;258(2):403–10. 31823059 10.1007/s00417-019-04515-8

[26] Chen Q, Ma RQ, Yi XQ, Gan L, Cheng Y, Zhang R, et al. Ultrasound biomicroscopy combined with doppler flow imaging in primary lacrimal canaliculitis. Ocul Immunol Inflamm. 2021;29(7–8):1403–9. 32275172 10.1080/09273948.2020.1738499

[27] Wawrzynski JR, Smith J, Sharma A, Saleh GM. OCT imaging of the proximal lacrimal system. Orbit. 2014;33(6):428–32. 25215411 10.3109/01676830.2014.949793

[28] Ye L, Yang BZ, Wang Y, Cheng HB, Peng Y, Zhang JX. Clinical features and therapeutic methods for inflammatory punctum disease. Trop J Pharm Res. 2017;16(8):2019–24.

[29] Timlin HM, Keane PA, Ezra DG. Characterizing congenital double punctum anomalies: clinical, endoscopic, and imaging findings. Ophthal Plast Reconstr Surg. 2019;35(6):549–52. 30865065 10.1097/IOP.0000000000001368

[30] Niedernolte B, Trunk L, Wolffsohn JS, Pult H, Bandlitz S. Evaluation of tear meniscus height using different clinical methods. Clin Exp Optom. 2021;104(7):763–9. 10.1080/08164622.2021.1878854 33689662

[31] Ang M, Baskaran M, Werkmeister RM, Chua J, Schmetterer L. Anterior segment optical coherence tomography. Surv Ophthalmol. 2018;63(3):293–321. 10.1016/j.preteyeres.2018.04.002 29635068

[32] Garaszczuk IK, Kałużny BJ, Malukiewicz G, Laudencka A, Podborączyńska-Jodko K, Wojtkowski M. Assessment of tear clearance rate with OCT. Optom Vis Sci. 2019;96(4):290–6.

[33] Kojima T, Dogru M, Ishida R, Goto E, Matsumoto Y, Kaido M, et al. Clinical usefulness of OCT for evaluating tear meniscus in dry eye. Invest Ophthalmol Vis Sci. 2013;54(13):8878–84.

[34] Wang J, Palakuru JR, Aquavella JV. Correlation between tear meniscus measurements by OCT and schirmer test. Cornea. 2008;27(8):867–71.

[35] Le Q, Xu J, Hong J, Sun X, Wei A, Zheng T, et al. Assessment of tear meniscus by OCT in dry eye. Eye (Lond). 2012;26(11):1349–53.22878449

[36] Qiu X, Gong L, Sun X, Jin H. Age-related variations of tear meniscus in healthy adults. Eye (Lond). 2010;24(3):600–4.19648904

[37] Shen M, Li J, Wang J, Ma H, Cai C, Tao A, et al. Upper and lower tear menisci in dry eye. Invest Ophthalmol Vis Sci. 2009;50(2):505–10. 19218609 10.1167/iovs.08-2704

[38] Savini G, Prabhawasat P, Kojima T, Grueterich M, Espana E, Goto E, et al. The challenge of dry eye diagnosis. Cornea. 2008;27(1):1–7. 19668387 10.2147/opth.s1496 PMC2698717

[39] Bandlitz S, Purslow C, Murphy PJ, Wolffsohn JS. Reliability of tear meniscus height measurements using different methods. Cont Lens Anterior Eye. 2019;42(4):431–6.

[40] Ahn SE, Jin LY, Ahn HB. Silicone tube intubation with conjunctival resection in refractory epiphora. Korean J Ophthalmol. 2018;32(6):438–44. 30549466 10.3341/kjo.2018.0044 PMC6288025

[41] Kashkouli MB, Beigi B, Murthy R. Fluorescein dye disappearance test: diagnostic accuracy for lacrimal drainage obstruction. Ophthal Plast Reconstr Surg. 2013;29(3):167–9. 23503058 10.1097/IOP.0b013e3182873b40

[42] Sasaki T, Asai T, Arai T. Punctal dilatation with perforated plugs for punctal stenosis. Jpn J Ophthalmol. 2015;59(6):433–9.

[43] Unlu M, Aslan B, Altinsoy HI, Sagdik HM, Okka M. Comparative results of snip punctoplasty techniques in acquired punctal stenosis. Eur J Ophthalmol. 2016;26(4):324–7.

[44] Thomas R, Walland MJ. Modified three-snip punctoplasty: results and complications. Aust N Z J Ophthalmol. 1990;18(4):409–13.

[45] Lloyd IC, Collin JR. Treatment of punctal stenosis with punctoplasty and intubation. Br J Ophthalmol. 1989;73(5):355–7.

[46] Ben Simon GJ, Joseph J, Lee S, Schwarcz RM, McCann JD, Goldberg RA. Simple punctal dilatation and curettage vs punctoplasty. Am J Ophthalmol. 2005;140(3):506–11.

[47] Bukhari A. Acquired punctal stenosis: etiological factors and associated findings. J Ocul Biol Dis Infor. 2010;3(1):17–9.

[48] Lee V, Bentley CR, Olver JM. Sutureless punctoplasty: a new technique. Br J Ophthalmol. 1998;82(1):95–7.

[49] Becker BB. Dacryology: the lacrimal drainage system. Am J Ophthalmol. 2003;136(4):687–94.

[50] Meyer DR, Linberg JV, Wobig JL, McCormick SA. Histopathology of lacrimal punctum in stenosis. Am J Ophthalmol. 1988;106(6):683–6.

